# Supplementary material for: Effects of Nutritional Interventions on Accuracy and Reaction Time with Relevance to Mental Fatigue in Sporting, Military, and Aerospace Populations: A Systematic Review and Meta-Analysis
Source: Int J Environ Res Public Health. 2021 Dec 28;19(1):307. doi: 10.3390/ijerph19010307 (PMC8744602; doi:10.3390/ijerph19010307)
Supplement: Supplementary file 1 [file ijerph-19-00307-s001.zip › ijerph-1466842-supplementary.pdf]

Risk of bias changes and exclusion details

Item removed	Rationale for removal
Q13<br>Were the staff, places, and facilities where the patients were treated, representative of the treatment the majority of patients receive?	These details are not typically reported in sport science, military, and aerospace studies. This item relates closer to *clinical* treatment of patients.
Q9<br>Have the characteristics of patients lost to follow-up been described?	Participants are not typically lost to follow-up in typically reported in sport science, military, and aerospace studies. This would only apply to with chronic nutrition interventions, and studies relevant to the topic of this meta-analysis were likely to be too acute/short-term to warrant including this item.
Q16<br>If any of the results of the study were based on “data dredging”, was this made clear?	Details are data dredging are rarely required in research relevant to this meta-analysis and were therefore not reported.
Q17<br>In trials and cohort studies, do the analyses adjust for different lengths of follow-up of patients, or in case-control studies, is the time period between the intervention and outcome the same for cases and controls?	Follow-ups are likely only relevant for parallel-group design studies with chronic nutrition interventions (e.g., 7- or 28-day supplementation). The majority of studies focused on acute interventions. Therefore, the lengths of follow-up are typically identical, thus, this item is largely irrelevant to sport science, military, and aerospace research.
**Item/s adapted**	**Rationale for adaptation**
Q5<br>"Are the distributions of principal confounders in each group of subjects to be compared clearly described? A list of principal confounders is provided.<br><br>Adaptation:<br>The list of principal confounders included cognitive ability, caffeine intake, energy drink usage, supplements, fitness, sleep	Provided clarity of confounders relevant to performance outcomes in sport science, military, and aerospace research.
